# Cellulose nanofibers (CNFs) in the recycling of nickel and cadmium battery metals using electrodeposition†

**DOI:** 10.1039/d3na00401e

**Published:** 2023-07-18

**Authors:** B. W. Hoogendoorn, O. Karlsson, X. Xiao, A. Pandey, S. E. Mattsson, V. Ström, R. L. Andersson, Y. Li, R. T. Olsson

**Affiliations:** a Department of Fibre and Polymer Technology, School of Engineering Sciences in Chemistry, Biotechnology and Health, KTH Royal Institute of Technology Teknikringen 56 114 28 Stockholm Sweden rols@kth.se; b SAFT AB Jungnergatan 25 572 32 Oskarshamn Sweden; c Department of Material Science and Engineering, School of Industrial Engineering and Management, KTH Royal Institute of Technology Brinellvägen 23 SE-100 24 Stockholm Sweden

## Abstract

Cellulose nanofibers (CNFs) were employed in the aqueous electrodeposition of nickel and cadmium for battery metal recycling. The electrowinning of mixed Ni–Cd metal ion recycling solutions demonstrated that cadmium with a purity of over 99% could be selectively extracted while leaving the nickel in the solution. Two types of CNFs were evaluated: negatively charged CNFs (a-CNF) obtained through acid hydrolysis (−75 μeq. g^−1^) and positively charged CNFs (q-CNF) functionalized with quaternary ammonium groups (+85 μeq. g^−1^). The inclusion of CNFs in the Ni–Cd electrolytes induced growth of cm-sized dendrites in conditions where dendrites were otherwise not observed, or increased the degree of dendritic growth when it was already present to a lesser extent. The augmented dendritic growth correlated with an increase in deposition yields of up to 30%. Additionally, it facilitated the formation of easily detachable dendritic structures, enabling more efficient processing on a large scale and enhancing the recovery of the toxic cadmium metal. Regardless of the charged nature of the CNFs, both negatively and positively charged CNFs led to a significant formation of protruding cadmium dendrites. When deposited separately, dendritic growth and increased deposition yields remained consistent for the cadmium metal. However, dendrites were not observed during the deposition of nickel; instead, uniformly deposited layers were formed, albeit at lower yields (20%), when positively charged CNFs were present. This paper explores the potential of utilizing cellulose and its derivatives as the world's largest biomass resource to enhance battery metal recycling processes.

## Abstract 2

Cellulose nanofibers (CNFs) were employed in the aqueous electrodeposition of nickel and cadmium for battery metal recycling.

## Graphical abstract

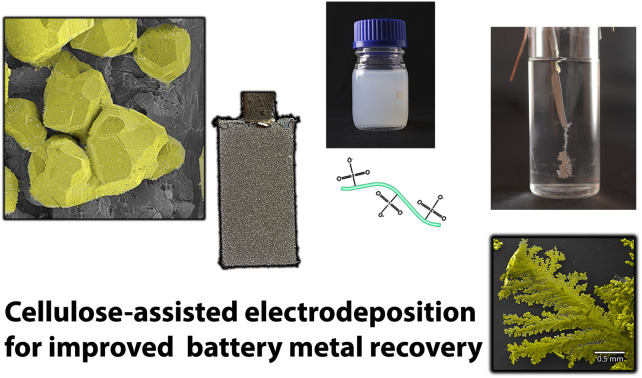

## 1. Introduction

Batteries are expected to be a cornerstone for energy storage as fossil fuels are phased out. The battery market has grown significantly in recent years, doubling its total market value to 120 billion USD in the last 5 years. This growth is expected to continue, with a projected annual demand growth of 25% until 2030.^1,2^ Meeting this development presents a challenge in terms of supplying battery manufacturers with the necessary metals at a rate that accommodates the battery production. Novel approaches to recycle already mined metals are therefore of the highest relevance.^3,4^

Aqueous acid leaching of the valuable metals from battery blackmass, followed by precipitation and crystallization, or electrodeposition and electrowinning, of the dissolved metal ions, has been suggested as the most effective method for the battery metal recovery.^5–9^ The leaching is beneficial due to the low energy consumption at low operating temperatures, and the limited use of toxic organic chemicals, which are key considerations in the metal recycling processes.^6,7,10,11^ However, the complex composition of leachates and how to perform the selective separation of the battery metals, presents a significant challenge.^8,11–13^ As the number of elements increase and the closer the metals are in their reactive nature, the more troublesome it becomes to avoid co-precipitation where multiple ions separate simultaneously.^8,11^ One strategy is to utilize chemical additives that allows for selective single-step recovery of each solvated metal ion. The drawback is that the additives used, such as quaternary ammonium salts, are highly toxic, resulting in additional steps and costs for recovering the additives themselves.^14,15^ A large-scale available biobased separation additive, providing a selective separation of the metal ions, would present a major step forward in the directions towards sustainable chemistry in the metal isolation from battery leachate solutions. Designing sustainable metal separation chemistry aligns with UN SDGs 9, 12, 13, 14, and 15, promoting innovation, responsible consumption and production, and ecosystem protection.^16^

Nanocellulose is a chemically stable, non-toxic and highly crystalline material, resistant towards acidic degradation at the pH levels (3-4) used in leaching metals from blackmass.^17^ Several reports show that crystalline nanocellulose can be chemically modified with great accuracy,^18–21^ providing the nanocellulose with an array of properties, which should open for selective interactions with different metal ions and its corresponding complexes.

Previously, biopolymers such as chitin/chitosan and proteins have been investigated in electrochemical operations due to their selective adsorption capacity.^22^ However, nanocellulose material has so far not been investigated as an additive during electrochemical recovery of battery metal ions even if several works have been published on the cellulose metal interactions in absorption/filtration applications.^23–25^

In this study, nanocellulose was used as a biobased electroactive additive during the electrodeposition recycling of nickel and cadmium (Ni–Cd) metal ions at industrial level leachate concentrations. A sulphate-based Ni–Cd system, mimicking the composition of industrial battery waste leachate when using sulphuric acid as active acid, was selected as a reference to evaluate the nanocellulose ability to influence the electrodeposition and isolation of solid nickel and cadmium. The effect of two types of cellulose nanofibres (CNF) were investigated, negatively charged (a-CNF) or positively charged (q-CNF) nanocellulose. a-CNF was obtained by acid hydrolysis, and q-CNF was obtained by modifying the a-CNF with quaternary ammonium salts. The aim was to investigate if the nanocellulose have the ability to interact with the charged metallic ion phase in solution (similar to as in the recycling of zinc battery metal ions^26^), providing recycling benefits of industrial value due to the inexpensive nature of cellulose. It is shown that cellulose can be used as an effective electroactive additive in nickel–cadmium separation to induce extensive dendritic growth of 99% pure metallic cadmium that allows for facilitated recovery of the toxic cadmium phase.

## 2. Experimental

### 2.1 Materials

Cadmium sulphate hydrate (3CdSO_4_ × 8H_2_O, ≥98%), nitric acid (HNO_3_, ≥65%), sulphuric acid (H_2_SO_4_, ≥98%), sodium sulphate (Na_2_SO_4_, ≥99%) sodium metasilicate (Na_2_SiO_3_, ≥98%), sodium carbonate (Na_2_CO_3_, ≥99%), sodium hydroxide (NaOH, ≥95%) and glycidyltrimethylammonium chloride (GTMAC, 90%) were all purchased from Sigma Aldrich. The nickel sulphate hexahydrate (NiSO_4_ × 6H_2_O, ≥98%) was bought from Supelco. Boric acid (H_3_BO_3_, ≥99%) was purchased from Alfa Aesar, while the hydrochloric acid (HCl, 37%) was purchased from Fischer Scientific. 0.5 mm thick titanium sheets (grade 1), graphite rods (Ted Pella INC, USA, diameter ≈3 mm) and epoxy adhesive (Henkel, Loctite EA3430) were all purchased from independent sellers. Aluminium silicate grit (*D* ∼ 0.25–0.42 mm) was used as a sandblasting medium and was purchased from Ahlsell AB (Sweden). The bacterial cellulose (BC) was grown from strains of *Acetobacter Xylinum* and was purchased from Monstra LLC (Thailand) as cubes with a volume of *ca.* 1 cm^3^. Milli-Q water (MQw, 18.2 MΩ, pH 7.0) was used to prepare all solutions. All chemicals were used as obtained.

### 2.2 Preparation of bacterial CNF

The isolation of bacterial CNF was inspired by a method previously developed by Hoogendoorn *et al.*, where the CNF had been obtained through acid hydrolysis using H_2_SO_4_.^26^

Prior to the acid hydrolysis, 2 kg of bacterial cellulose (BC) cubes were prepared by soaking the cubes in water for 5 days. The BC was exposed to 10 vol% NaOH at 100 °C for 10 min before being rinsed for 24 h in water. The pre-treatment assured the full removal of residual growth medium, bacteria, and other organic contaminations. A blender (Blendtec 625) was used to shred the cubes into a slurry, which was partially dewatered by compressing the material using a polyamide mesh (pore size: 46 μm, SEFAR). The subsequent acid hydrolysis was performed by adding the compressed BC into 1 L of a 50 vol% H_2_SO_4_ -solution heated to 60 °C. The acid hydrolysis proceeded for 4 h under stirring before being quenched and terminated by the addition of 1 L of MQw. The hydrolysed cellulose was centrifuged at 11 000 × *G* for 10 minutes, 3 times, which included two intermediate exchanges of the supernatant with MQw. The obtained dispersion was dialysed for 5 days using dialysis tubing (MWCO = 100 kDa), until a neutral pH had been obtained. The resulting CNF (referred to as a-CNF in this study) had a solid content of 0.97 wt%. The surface charge of the CNF was determined on triplicate samples through polyelectrolyte titration using polydiallyldimethylammonium chloride (P-DADMAC) as titrant.

### 2.3 Cationic functionalization of bacterial CNF

Quaternized CNF (q-CNF) was prepared from the acid hydrolysed CNF (a-CNF). Prior to the functionalization, 100 ml of the a-CNF dispersion was concentrated up to a solid content of *ca.* 1.7 wt% through centrifugation at 33 000 × *G*. 1 ml of an aqueous NaOH solution (0.33 g ml^−1^) was added to the dispersion to activate the hydroxyl groups before the functionalization. The alkaline dispersion was conditioned for 45 min whilst being heated to 65 ± 0.5 °C. The reaction was performed by adding 25 g of glycidyltrimethylammonium chloride (GTMAC) to the dispersion, allowing it to proceed at 65 ± 0.5 °C under vigorous stirring for 8 h. The reaction was quenched by cooling the solution in an ice bath and adding cold HCl (aq., 0.5 M). The mixture was purified through dialysis until a neutral pH and a conductivity <15 μS cm^−1^ had been reached for the dispersion. The properties of the q-CNF, in terms of dimensions and surface charge, were determined using the same methods as for the a-CNF. The surface charge was likewise determined through the same methodology as for a-CNF, but with the anionically charged potassium polyvinylsulfonate (KPVS) as titrant instead of the cationically charged P-DADMAC.

### 2.4 Cathode preparation

**Fig. 1:**
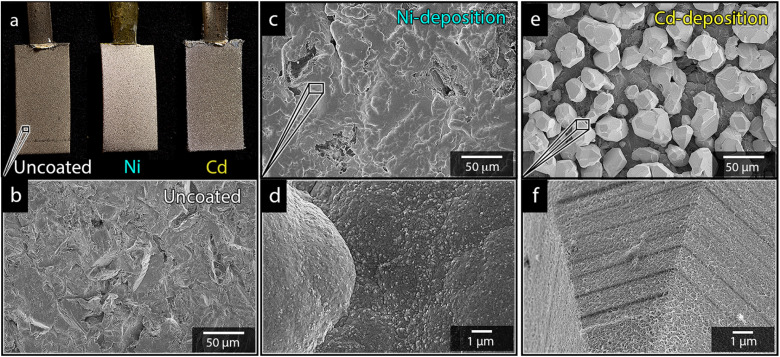
Photograph showing the appearance of an uncoated, a nickel coated, and a cadmium coated Ti-electrode (a). Micrographs of an uncoated electrode (b) and electrodes deposited with nickel (c and d) and cadmium (e and f). All electrodes were deposited at 40 °C, 3.5 V and metal-ion concentrations of 0.44 mol L^−1^.

Paddle-shaped titanium cathodes (see Fig. 1a) were prepared as previously described by Hoogendoorn *et al.*^26^ The preparation method resulted in 8 cm long titanium strips, with a bottom section with the dimensions of 1 × 2 cm, see Fig. 1. The rectangular section on which the electrodeposition was made, was sandblasted using the aluminium silicate to provide the electrodes with uniform and identical surface textures (Fig. 1a and b). The active surface of the electrodes was limited to the 1 × 2 cm sections by coating the boundary between the rectangular section and the narrow upper part of the cathodes with an epoxy adhesive.

### 2.5 Electrodeposition of nickel and cadmium

The electrodeposition was carried out in a reactor with dimensions of 6 cm × 17 cm × 10 cm. For the single metal-ion protocols, the aqueous electrolyte was composed of the respective sulfate-based metal salt at a concentration corresponding to 0.44 mol L^−1^ of Ni^2+^ or Cd^2+^, sodium sulfate (12 g L^−1^) and boric acid (40 g L^−1^). For the mixed systems, the added metal salts corresponded to the addition of varying ratios of Cd- and Ni-ions that always lead to a total metal ion concentration of 0.44 mol L^−1^ (see Table S1†), *i.e*., the same total concentration of metal ions as in the cases of the separate metal ion deposition. The electrolytes were prepared 12 h before the electrodeposition to ensure that an equilibrium state of the metal ionic species was reached. All electrolytes showed a pH of 3.7 ± 0.1. All experiments were started by adding 0.5 L of the electrolyte to the reactor and heating it to 40 ± 1 °C. The electrolyte was further purged with argon for 30 min prior to initiating the electrodeposition. During the deairing procedure, the titanium cathodes were pre-treated separately by first submerging them for 10 min in an aqueous alkaline bath (40 g L^−1^ NaOH, 25 g L^−1^ NaCO_3_, 25 g L^−1^ Na_2_SiO_3_) heated to 90 ± 1 °C, before pickling them for 10 min in Aqua Regia. The procedure ensured that the cathodes were fully degreased and removed of any oxide species prior to the electrodeposition. The cathodes were fixed in the middle of the reactor while two graphite rods, used as anodes, were placed on each side of the reactor. The electrodeposition experiments were performed at potentiostatic conditions at a voltage of 3.5 V for 10 min. The experiments were carried out without CNF and with 0.5 g L^−1^ CNF dispersed in the solution (0.05 wt%). After the experiments, the cathodes were rinsed with MQw and dried at room temperature. The yields were determined by weighing the cathode before and after the electrodeposition and through inductively coupled plasma atomic emission spectroscopy (ICP-AES), as described in Section 2.6.

### 2.6 Characterization

#### 2.6.1 Microscopy

Scanning electron microscopy (SEM) and scanning transmission electron microscopy (STEM) was performed using a Hitachi S-4800 SEM. Prior to capturing images through SEM, the specimens were coated with platinum/palladium for 40 s using a Cressington 208RH sputter coater. During the STEM, no coating was applied. While micrographs obtained through SEM were taken an acceleration voltage of 5 kV and an emission current of 10 μA, the STEM-micrographs were captured at an accelerating voltage of 16 kV and an emission current of 10 μA. For each deposition, micrographs were taken of several sections of the electrode to confirm that the morphology was representative for the entire electrode, *i.e.* the entire electrode was placed in the SEM. Likewise, several samples were observed using microscopy to confirm that the morphology was representative across multiple samples.

#### 2.6.2 Elemental analysis

Energy dispersive X-ray spectroscopy (EDX) was performed on all samples using the SEM specified in the microscopy section. The EDX-mapping was made at an acceleration voltage of 17 kV and an applied current of 10 μA. Every mapping was performed for 10 min.

The quantitative compositional analysis of the deposited material was performed on all samples using a SpectroBlue TI ICP-AES. The deposits were dissolved by exposing the coated cathode to 20 ml of 5 vol% HNO_3_ (aq.) for 24 h. The dissolved material was diluted to concentrations appropriate for the ICP-AES measurements. The compositional analysis was determined using always a minimum duplicate (≥2) of samples for the depositions made without the presence and in the presence of CNF.

#### 2.6.3 X-ray diffraction

The crystalline structure of the electrodeposited cathodes as well as freeze-dried samples of the different varieties of CNF was investigated using a PANalytical X'pert PRO X-ray diffractometer. A Cu K α-radiation source was used at a voltage and current of 45 kV and 40 mA. The measurements were performed at an angular interval from 5° to 80° for the deposited cathodes, and 5° to 60° for the CNF-samples. All measurements were done at a step size of 0.05° and a wavelength of 0.154 nm.

The crystallinity of the CNF-samples was determined through peak-deconvolution, as described by Park *et al.*^27^ The different cellulose peaks were deconvoluted using gaussian curve fitting (Fig. S1†) before using eqn (1) below to determine the crystallinity.1
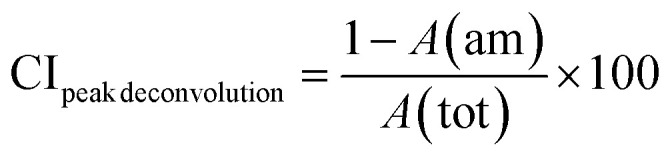


In eqn (1), CI_peak deconvolution_ stands for the crystallinity in percent, while *A*(am) corresponds to the area of amorphous halo found in the cellulose diffractogram at around 2*θ* = 20°. *A*(tot) is the combined area of the amorphous halo and the crystalline peaks defined as the (101)-, (101̄)-, (002)-, (021)- and (040)-lattice planes, as according to the convention used by Park *et al.*^27^ The crystallite size of the deposits was determined by using the Scherrer equation (eqn (2)).2
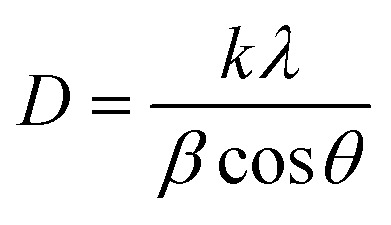
where *D* represents the average crystallite size, *k* is the shape factor, *λ* the wavelength, *β* the peak width at half maximum intensity, and is the Bragg angle.

#### 2.6.4 Fourier-transform infrared spectroscopy (FTIR-spectroscopy)

The functional groups of the a-CNF as well as the q-CNF functionalized with GTMAC were characterized using a PerkinElmer Spectrum 100 spectrometer on samples freeze-dried for 48 h prior to the spectroscopy. The instrument was fitted with an MIR TGS detector and the measurements were performed 16 times within an interval of 600–4000 cm^−1^ at a scan rate of 1 cm^−1^ and resolution of 4 cm^−1^.

## 3. Results and discussion

### 3.1 Electrodeposition of Ni or Cd in single-metal electrolytes

**Fig. 2:**
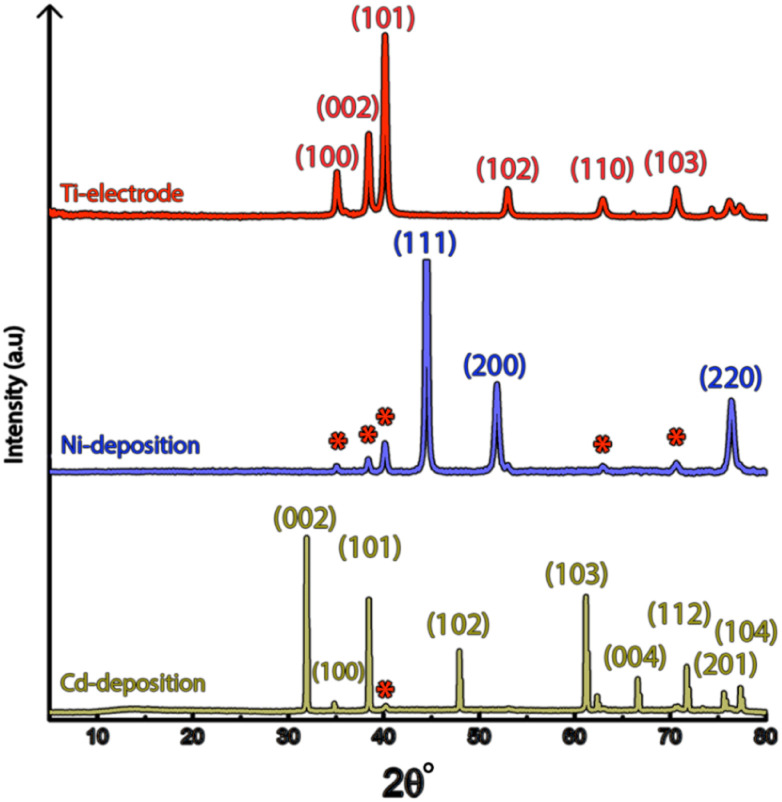
(a) X-ray diffractograms of the titanium electrode prior to electrodeposition and after 10 min of electrodeposition of nickel and cadmium. respectively. The red symbols indicate the peaks given by the titanium electrode.

Fig. 1a shows the nickel and cadmium coated cathodes after 10 min of electrodeposition compared to the uncoated titanium cathode. The micrograph in Fig. 1b demonstrates that the uncoated electrode had a rough surface texture due to the abrasive aluminium silicate treatment carried out on all electrodes before electrodeposition experiments. The usage of the aluminium silicate treatment was made to reduce the presence of imperfections and differences between the electrodes that could otherwise introduce artifacts during the electrodeposition and complicate sample comparison.^26^Fig. 1a and c–f show that regardless of the specific metal ion, the electrodeposition always resulted in an even coverage of the electrode surfaces with brightly coloured shiny deposits. The deposited cadmium (Cd), however, showed a significant growth of 25 to 100 μm dodecahedral particles structures evenly spread over the cathode surface, Fig. 1e, whereas the nickel (Ni) formed a uniform and coherent coating in absence of any facetted particles (Fig. 1c). The uniform deposition of nickel was consistent with a non-preferential deposition (of the incoming Ni-ions) onto the Ti-substrate, or the already deposited Ni material, as shown in Fig. 1c and d. The formation and growth of the cadmium particles combined with regions without deposition is indicative of a growth pattern generally referred to as Volmer–Weber growth, which stands in contrast to layer-by-layer deposition resulting in an even surface coverage.^28–30^ The X-ray diffractogram, shown in Fig. 2 confirmed that both deposits were metallic, although the morphological differences verify that the mechanisms with which they deposit are distinctly different. The diffractograms were used to determine the crystallite size *via* the Scherrer equation. The crystallite sizes, shown in Table S2,† showed that the granular Ni-deposit consisted of smaller crystallites, with an average size below 20 nm, while the faceted Cd-deposits had a consisted of crystallites that were significantly larger, with an average size of *ca.* 60 nm.

**Fig. 3:**
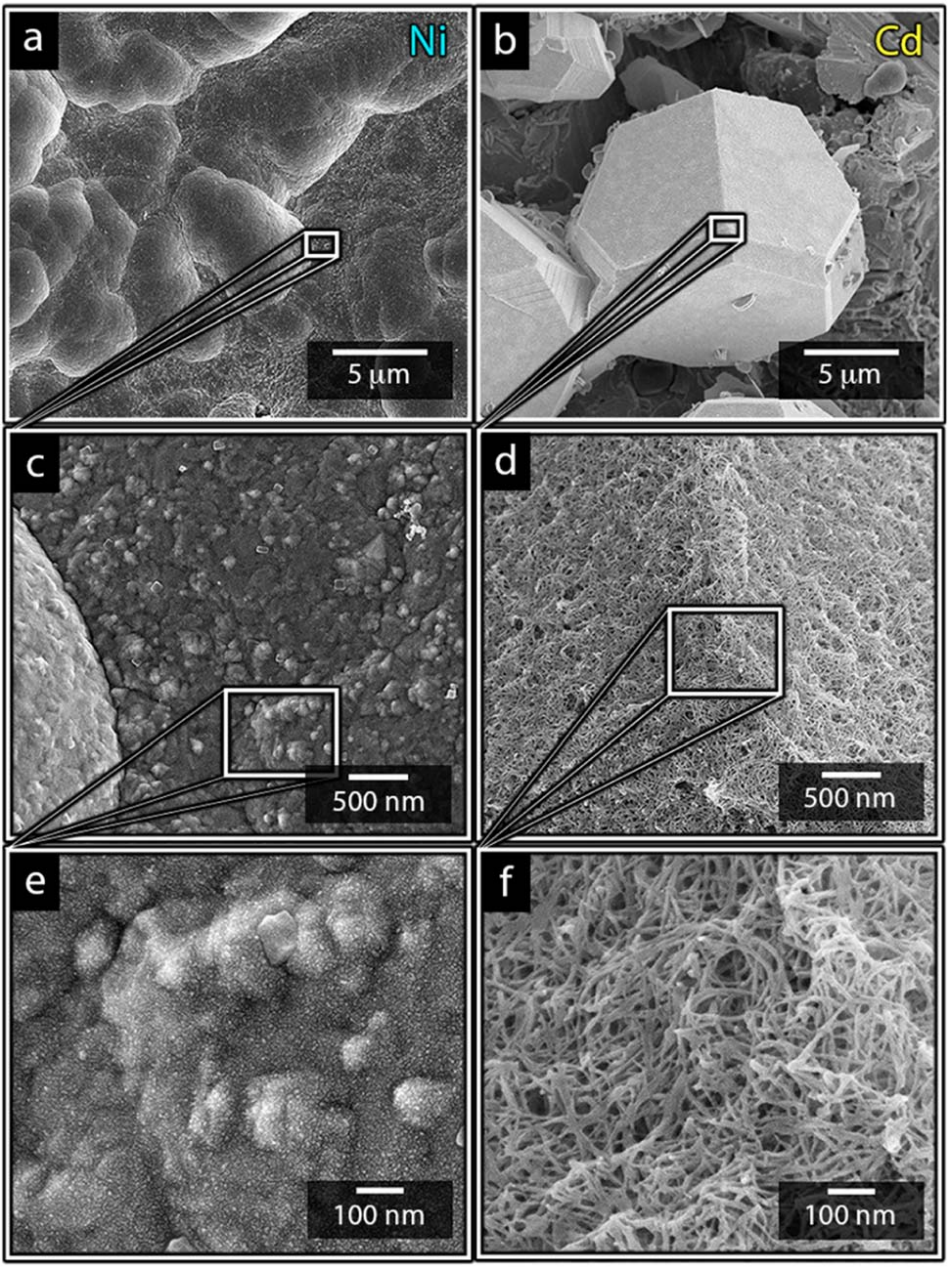
Micrographs of nickel (a, c and e) and cadmium (b, d and f) that have been electrodeposited at 40 °C for 10 min at a metal-ion concentration of 0.44 mol L^−1^. The inset reveals the nanostructures building up the surfaced of the deposited structures.

Fig. 3 highlights that the nickel deposits consisted of a dense assembly of nanograins (Fig. 3a, c, and e), whereas the truncated surfaces associated with the cadmium particles were covered by *ca.* 10 nm thick and continuous cadmium nanofilaments (Fig. 3b, d and f). The fact that the cadmium particles had a strongly faceted appearance, despite an exterior consisting of randomly oriented filaments, suggests that the filaments concealed a solid and denser faceted metallic core.

It is noteworthy is that the faceted Cd-structures shared similarities with previously reported electrodeposited structures of zinc, which similarly have been shown to grow faceted structures in agreement with Volmer–Weber growth under electrical fields.^26^ The formation of cadmium-based filaments has, however, not been reported in connection to acidic electrodeposition although the morphology shares a strong resemblance to cadmium oxide or hydroxide nanowires (fibrils).^31–33^ Fig. S2† shows that the electrodeposited cadmium filaments did not display any morphological changes over the long-time storage of one month. Additionally, the X-ray diffractogram in Fig. 2 does not reveal any presence of oxides or hydroxides on the electrodes, which are predominantly characterized by additional peaks around 2*θ* = 18°,^32^ indicating that the presence of phases other than the metallic cadmium were not significant to be detected by conventional crystallographic measurements.

Overall, the difference in the deposition mechanisms appeared to be governed by the nature of the metal ions diffusion in the solutions. Here, the inability of nickel to grow visible protruding structures suggests that the formation of nickel was less affected by mass transfer phenomena under the deposition conditions set during this study, most likely due to a more limited charge transfer kinetics. A slow charge transfer kinetics would be supported by a slower deposition rate and a more even distribution of deposits since a slower charge transfer allows the mass transfer to be fast enough to avoid rapid depletion of ions at the electrode surface or uneven distribution of metal ions along the electrode surface.^34^

Table 1 shows the separate nickel and cadmium deposition yields after 10 min of electrodeposition. While both metals were revealed to deposit a significant amount of material within the reaction time, the amount of deposited nickel was always smaller (0.79 mol m^−2^) compared to the cadmium (1.01 mol m^−2^) for the same potential, deposition times and metal ion concentrations.

**Table 1:** Absolute yields of deposited nickel and cadmium after 10 min of electrodeposition in the single-metal protocols in units of mol m^−2^, without the presence of CNF. The measurements are obtained as described in Section 2.5

E-deposition metal (s)	Deposition by weight^a^ (g m^−2^)	Deposition by moles^a^ (mol m^−2^)	Deposited by ICP-AES^a^ (mol m^−2^)
Nickel	46 ± 5.5	0.79 ± 0.09	0.82 ± 0.14
Cadmium	113 ± 11	1.01 ± 0.10	1.05 ± 0.08

a Deposited amounts normalised to the surface area of the electrode at *t* = 0.

The values shown in Table 1 were obtained by weighing the samples before and after the electrodeposition experiments (as according to Section 2.5). These values were shown to agree with the yields obtained through ICP-AES (see Table 1), confirming the validity of the weighing of the cathodes to determine the yield. It deserves to be underlined that these depositions constitute only a small fraction of the total mass of Ni- or Cd in the electrolytes (<0.1%), *i.e.,* none of the effects of electrodeposition could be attributed to a depletion of metal ions in the bulk electrolyte solution.

### 3.2 Making differently charged cellulose nanofibers (CNF)

**Fig. 4:**
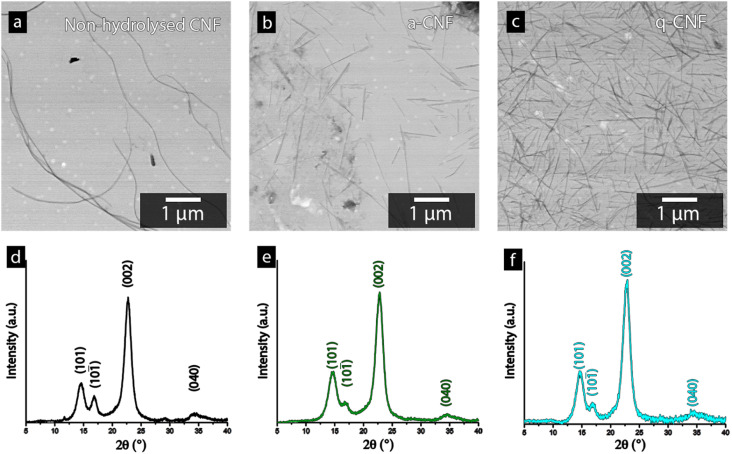
STEM-micrographs of the cellulose nanofibers (CNF) obtained before the acid hydrolysis (a), a-CNF(b) and q-CNF (c). The graphs show the corresponding diffractogram for the CNFs before the hydrolysis (d) the a-CNF (e) and the q-CNF (f).

Fig. 4a–c shows the STEM-micrographs of the bacterial cellulose prior to and after the acid hydrolysis (a-CNF), as well as after the quaternization protocol (q-CNF). The hydrolysis resulted in a significant decrease in the length of the nanofibrils from >5 μm to *ca.* 500–600 nm (Table 2). The decrease in length with the acidic hydrolysis was explained by the acidic conditions resulting in the cleavage of the glycosidic bond linking the glucose units, eventually cleaving the entire fibrils.^35,36^ The dimensions of the individual nanofibrils were however the same after the additional quaternization reaction had been carried out (q-CNFs). The average values furthermore agreed with the previously reported values for this particular hydrolysis protocol.^26^ The X-ray diffractograms shown in Fig. 4d–f followed the characteristic pattern of cellulose; the peaks corresponding to the (101), (101̄), (002) and (040) were the most pronounced peaks. The diffractograms were used to derive the crystallinity as described in Section 2.6, see Table 2.

**Table 2:** Properties of the two types of CNF used during the electrodeposition in terms of dimensions and surface charge

Cellulose type	Average length^a^ (nm)	Crystallinity	Surface charge (μeq. g^−1^)
Non-hydrolysed CNF	>5000^a^	73	−30 ± 10
a-CNF	550 ± 45	74	−75 0
q-CNF	560 ± 40	73	+85 ± 10

a Due to the vast length and the tendency to form aggregates prevented accurate statistical determination average length.

The ability to accurately derive the amorphous contribution of cellulose, avoiding the inclusion of crystalline contributions concealed by the amorphous cellulose and the background, as well as obtaining accurate results for different allomorphs', have been questioned.^37^ For clarity, Fig. S1† therefore shows the typical appearance of the deconvoluted peaks used to determine the crystallinity of the bacterial nanocellulose used in this study. Bacterial cellulose predominantly consist of the I α-allomorph of cellulose as compared to the I β kind more common in plant-based cellulose, which brings an additional caveat when comparing the crystallinity of cellulose in other works.^38,39^ However, the intensity of the (002)-peak for all diffractograms always exceeded 1500 counts, assuring that noise interference had a minimal impact on the determination of the crystallinity in this work. Despite these factors, it needs to be emphasised that the absolute crystallinity values should be considered as estimates due to the inherent uncertainties in the method. Furthermore, due to the consistency when deconvoluting the peaks and the high intensity of the diffractogram, the values conclude that the crystallinity remains constant after the hydrolysis as well as after the quaternization, see Table 2.

Table 2 further shows that the net surface charge of q-CNF after the quaternization reaction was +85 μeq. g^−1^ and similar to that of a-CNF with −75 μeq. g^−1^ due to its cationic nature. The positive charges formed as a consequence of the grafted quaternary ammonium groups, as opposed to the negatively charged groups present on the surface of a-CNF. The negative charges, in turn, were a result of the substitution of sulfate half-esters during the acid hydrolysis (see Fig. S3–S4† for an illustration of the chemical structure of the two CNF types).^40^

**Fig. 5:**
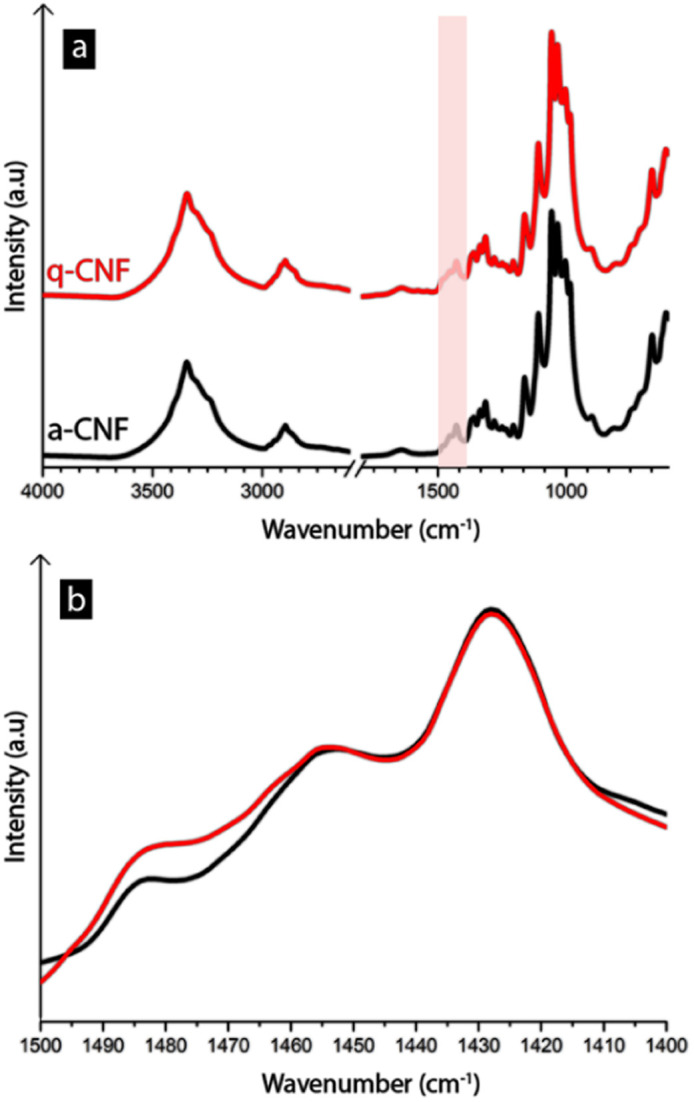
FTIR-spectra of freeze-dried sampled of a-CNF and q-CNF. The arrows in (a) highlights the region that is shown in (b), in which the height increase around 1485 cm^−1^ correspond to the methyl groups present in the quaternary ammonium group.

The quaternary ammonium groups were further confirmed using FTIR-spectroscopy (see Fig. 5), where a small peak had appeared around 1475–1485 cm^−1^, which corresponds to the trimethyl-groups present in the quaternary ammonium group.^41–43^

Previously, quaternary ammonium salts have been used to affect electrodeposition in the case of copper, manganese, and nickel due to their ability to adsorb to the electrode surface, resulting in deposition inhibition.^15,44,45^ However, the toxicity of quaternary ammonium salts has raised questions regarding their feasibility as an additive in industrial procedures.^14^ Having the ammonium group covalently bound to nanofibers is advantageous since it allows for the advantageous properties of the quaternary ammonium to maintain while preventing it from dissolving in the liquid so it can be collected and recycled *via* filtration or sedimentation.

### 3.3 Electrodeposition of nickel or cadmium in the presence of nanocellulose

**Fig. 6:**
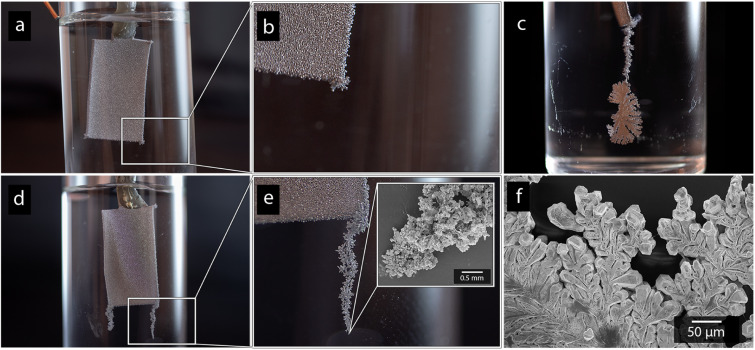
Photographs of electrodes deposited with cadmium without the presence of CNF (a and b) and in the presence of a-CNF (c–e) after 10 min deposition. Image (c and e) highlights the appearance of the protruding structures growing at the edges of the electrode, with the inset in (e) and the micrograph in (f) showing SEM micrographs of protruding dendrite structures in high resolution.

Fig. 6 shows photographs of the cadmium electrode depositions made in absence of CNF (Fig. 6a and b), and with a-CNF dispersed in the electrolyte (Fig. 6c–f), after 10 min of deposition time. The presence of a-CNF resulted in a formation of protruding cadmium dendrites at the electrode edges, reaching up to 10 mm or more in length, see Fig. 6c–f. The strong formation of cm-scale dendrites suggested that the deposition became increasingly limited by mass transfer when the 0.5 g L^−1^ (0.05 wt%) CNF was present in the solution (Fig. 6c–e), similar to previously reported zinc-deposition.^26^ The associative interactions between cellulose and metal ionic species were therefore argued to decrease the mobility of the metal ionic species during the deposition.^26^ However, the growth of the protruding dendrites was similar as the concentration was increased to 1 g L^−1^ (0.1 wt%), and it always occurred at areas where the electrical field gradient is the highest (corners, edges and protruding structure *etc.*), suggesting that the mass transfer was dominated by migration in relation to diffusion.^46^ A rapid migration, the movement of charged ions due to the presence of electrical potential gradients, will lead to the movement of the metal ions being more affected by the strength of the electric field, as opposed to concentration gradients.^47–49^ The shape of the dendritic structures could, however, vary as more extended protrusions formed with small differences in growth pattern, compare Fig. 6c and e. Smaller concentrations of 0.25 g L^−1^ (0.025 wt%) were also evaluated and was likewise found to initiate dendritic growth, but the 0.5 g L^−1^ was providing an optimal combination of initiating significant dendrite growth while avoiding challenges with excessively viscous CNF suspensions. In fact, a 2 g L^−1^ (0.2 wt%) solution could not be used due to practical reasons, *e.g.* showing a gel-like behavior.

The formation of dendritic structures had a marked effect on the cadmium yields (shown in Table 3) by providing greater active surface area for subsequent deposition, resulting in increased yields up to ≈15 wt% over the 10 min deposition time. The formation of protruding cadmium structures was however exclusive to the deposition of cadmium, which explained why a similar increase in yield did not occur when nickel was being deposited (Table 3). Interestingly, the dendrite formations were induced for the cadmium regardless of if the CNF was present as a-CNF or q-CNF in the electrolyte, suggesting that the charge of the cellulose had no, or little effect, on the cadmium deposition. In contrast, a positively charged cellulose (q-CNF) resulted in a substantially smaller deposition of the nickel, which, besides showing that the surface charge only affected Ni-deposition, also suggest that the nickel was present in a form that could interact more strongly with the surface of the CNF, see further Section 3.4. This lack of visible protrusions during Ni-deposition in the presence of CNF (see Fig. S5†) suggests that the slower charge transfer of nickel reduces the resulting effect of nanocellulose since the electrodeposition, despite of the decreased mass transfer rates, still is in the regime of charge transfer control. The formation of protruding deposits is also argued to be less enforced in the case of nickel due to the less pronounced preference to follow Volmer–Weber growth.^28,29^ This is different to the case of cadmium, where the diffusion of metal ions to the electrode is the rate-limiting step as opposed to the charge transfer occurring at the electrode surface.^29,34,50^

**Table 3:** Absolute yields of deposited nickel and cadmium after 10 min of electrodeposition in the single-metal protocols in units of mol m^−2^, in the presence of 0.5 g L^−1^ of a-CNF or q-CNF

Reaction type	Absolute yields of deposited metals (mol m^−2^)	
	Ni-deposition	Cd-deposition
No CNF	0.79 mol m^−2^ (from Table 1)	1.01 mol m^−2^ (from Table 1)
a-CNF	0.81 mol m^−2^	1.11 mol m^−2^
q-CNF	0.63 mol m^−2^	1.17 mol m^−2^

**Fig. 7:**
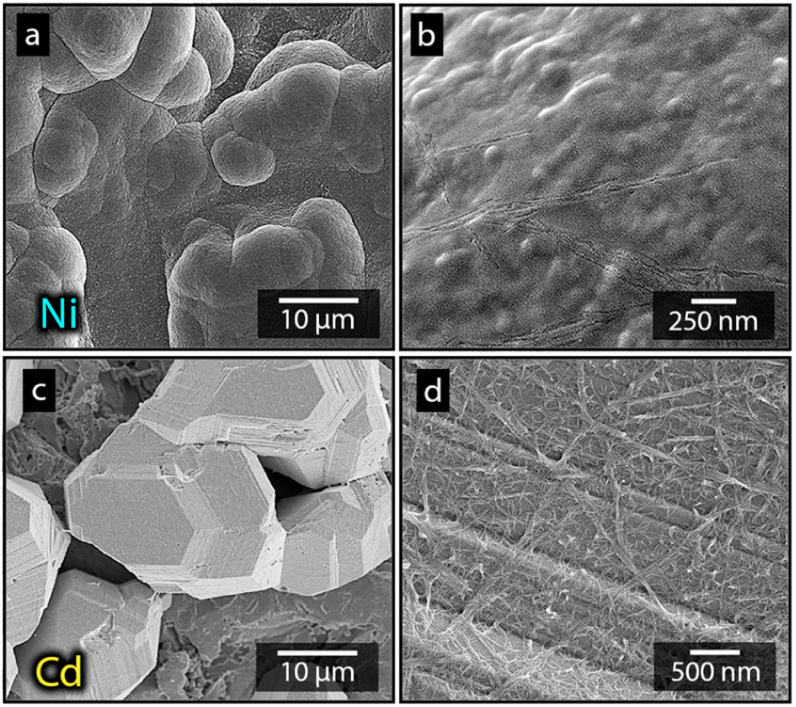
Micrographs showing electrodeposited nickel (a and b) and cadmium (c and d) after 10 min of electrodeposition, at 3.5 V, in the presence of a-CNF (b and d).

Fig. 7 shows the more in detail microscopy analysis of the nickel and cadmium deposits in the absence and in the presence of the nanocellulose. Fig. 7a and b show that the compact Ni-deposits always occurred even in the presence of nanocellulose, regardless of the net surface charge of the cellulose. It was also apparent from the higher resolution microscopy that the facetted cadmium deposits were obtained in the presence of CNF as well. The CNFs could clearly be observed as a surface feature on top of the Cd-deposits (Fig. 7d). An incorporation of CNF into the metallic phase was never observed. It was also noted that the size of the particles varied insignificantly compared to when no nanocellulose was present, compare Fig. 7c, 1e and 3b. In summary, it was concluded that the dendritic growth was not associated with any changes in the crystalline structure of the metallic cadmium (see diffractogram in Fig. S6†), or morphological changes of the individual cadmium particles. The effects of the CNFs on the cadmium deposits were instead observed on a macroscopic scale by functioning as a catalyst for dendrite formation, which was shown to have an effect on the cadmium yields.

### 3.4 Electrodeposition of the nickel and cadmium from their multi-metal electrolyte at industrial concentration

**Fig. 8:**
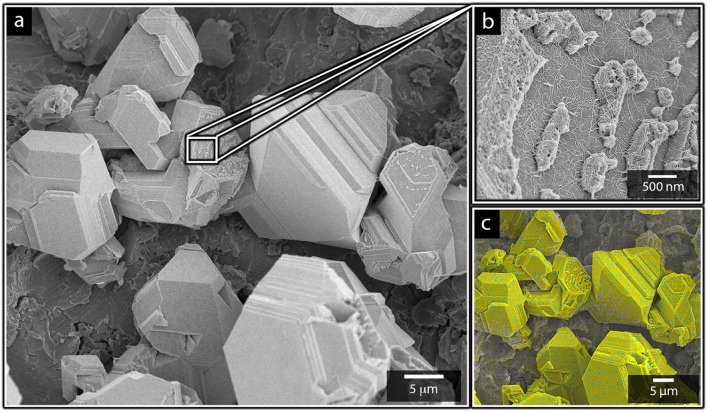
(a and b) Micrographs of cathodes exposed to 10 min of electrodeposition in electrolyte containing equimolar amounts of nickel and cadmium (0.22 mol L^−1^). (c) EDX-mapping of the electrodeposited surfaces, showing the EDX-readings of nickel (blue) and cadmium (yellow).

Fig. 8 shows the cathode after 10 min of electrodeposition in the electrolyte containing equimolar amounts of Ni- and Cd-ions at concentrations 0.22 M (total 0.44 M). Although the conditions used in the single metal depositions (Section 3.2 and 3.3) resulted in significant amounts of deposited material for both metals individually (Ni and Cd, see Tables 1 and 3), the same conditions for the mixed metal system resulted in only deposition of cadmium without a trace of nickel deposited on the electrode surface, see Fig. 8a–c. The selective deposition of cadmium in the presence of nickel was previously reported by Rudnik *et al.*, Mohanty *et al.* and Hazotte *et al.*^7,8,51^ The reports suggests that minimal amounts of Cd-ions hinder the electrodeposition of nickel, despite being present at much higher concentrations than the cadmium. The proposed explanation being the cadmium ions adsorbing onto the electrode, subsequently polarizing the cathode, with the consequence of blocking the active sites of the cathode surface.^51^

The micrographs also demonstrate that the characteristic morphology of the truncated faceted cadmium particles still remains as a prominent feature (Fig. 8a), even in the presence of nickel. The same was shown to apply for the cadmium-based nanofilaments, as shown in Fig. 8b.

**Fig. 9:**
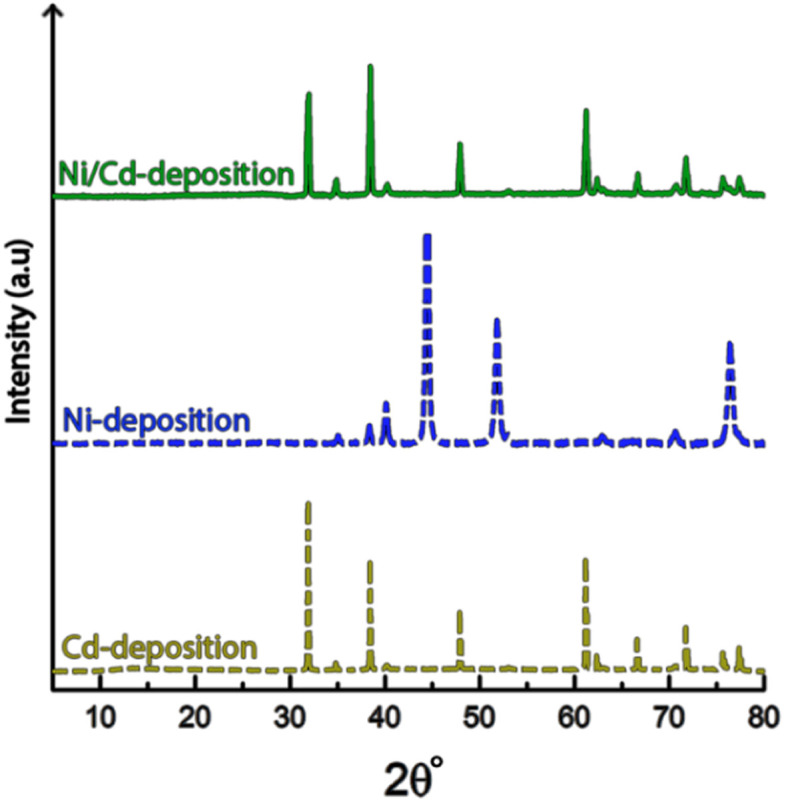
X-ray diffractogram of cathodes exposed to 10 min of electrodeposition, at 3.5 V in multi-metal electrolytes containing both nickel and cadmium (top), as compared to electrolytes containing only nickel (middle) and cadmium (bottom).

Fig. 9 shows the X-ray diffractogram of an electrode exposed to electrodeposition in a mixed electrolyte compared to the diffractograms obtained from electrodes deposited in Ni-and Cd-electrolytes, respectively.

The similarity between the mixed metal diffractogram and the Cd-diffractogram as well as the lack of any characteristic Ni-peaks confirms that only metallic cadmium had been deposited, which further established the highly selective deposition behaviour when both metal ions are present in the electrolyte.

### 3.5 CNF-assisted deposition of Cd in multi-metal electrolytes and quantitative evaluation of electroactive CNF performance

Table 4 shows the amount of the deposited nickel and cadmium during the mixed-metal deposition with, as well as without the presence of CNF. The measurements quantitatively confirms that the electrodeposition protocol is highly selective towards depositing cadmium, despite having nickel ions present at equimolar conditions. It is revealed that amounts of deposited cadmium always exceeded 1 mol m^−2^, while the amount of deposited nickel was below the detection limit. When described in relation to the surface area of the cathode, this corresponds to <0.005 mol m^−2^. The highly selective deposition of cadmium was present regardless of if a-CNF or q-CNF was present in the electrolyte, never showing an amount of deposited nickel above the detection limit. It is noteworthy that whereas both the nickel and the cadmium metal ions are considered weakly acidic metal ions in aqueous solutions, the nickel ions are slightly more prone to forming hydroxides at pH 3–4 than the cadmium ions, possibly affecting its interactions towards the CNF and the electrode.^24,52,53^

**Table 4:** Absolute yields of deposited nickel and cadmium after 10 min of electrodeposition in the multi-metal protocols and in the presence of 0.5 g L^−1^ of a-CNF or q-CNF

Reaction type	Absolute yields of deposited metals (mol m^−2^)’	
	Ni-deposition^a^	Cd-deposition
a-CNF	<0.005 mol m^−2^	1.01 ± 0.05 mol m^−2^
q-CNF	<0.005 mol m^−2^	1.12 ± 0.08 mol m^−2^

a The concentrations of the dissolved deposits were below the detection limit of the instrument. In this case that limit corresponds to *ca.* 0.005 mol m^−2^.

**Fig. 10:**
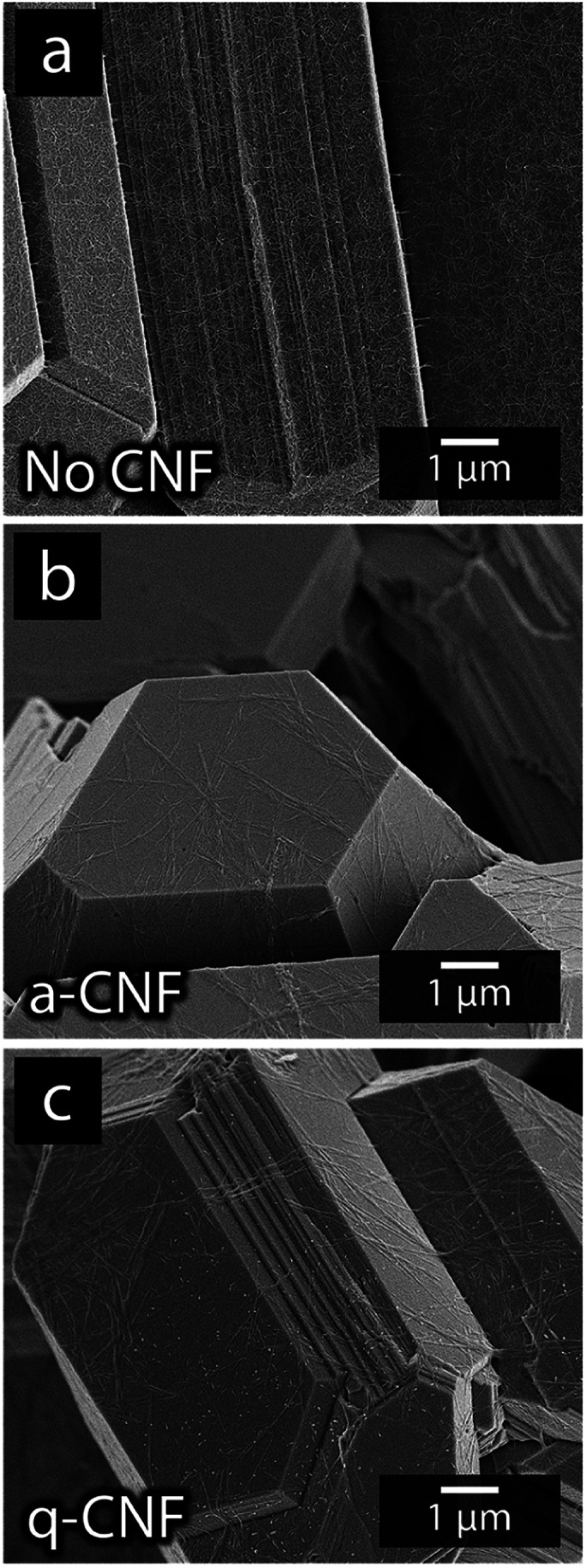
Micrographs of the deposits formed using mixed-metal electrolytes without the presence of CNF (a), in the presence of negatively charged a-CNF (b) and positively charged q-CNF (c).

Fig. 10 shows the appearance of the electrodeposited material obtained after the simultaneous nickel and cadmium deposition without any CNF present (a), in the presence of a-CNF (b) and q-CNF (c). The morphology of the deposits in the presence of a- and q-CNF is shown to remain similar to the material deposited without CNF on a nanoscale. From Fig. 10 it is apparent that the function of the CNF was primarily to induce dendritic growth since the truncated areas remained unaffected in dimensions and shapes by the presence of the nanocellulose. The a-CNF and q-CNF can be seen in Fig. 10b and c, respectively, as covering the faceted metallic dodecahedrons. However, the only visible effect on the metal deposits was that the cadmium filaments disappeared in the presence of CNF (see Section 3.4). The observations that q-CNF only affected nickel deposition during single metal deposition, while the deposition of cadmium remained the same for a-CNF and q-CNF, and that no measurable amounts of Ni-deposits could be detected with ICP-AES in mixed electrolytes containing equimolar amounts of Ni- and Cd-ions, was used as motivation for conducting the continued study on mixed electrolytes on solely the a-CNF.

Table 5 shows to which extent the CNF could facilitate the dendritic growth for different compositions of the multi metal electrolyte, with cadmium concentrations ranging from ∼10% (0.04 M) to ∼90% (0.4 M), approaching the 0.44 M cadmium concentration that resulted in only truncated and facetted cadmium particles in the single metal deposition experiment (see Section 3.1 and 3.3).

**Table 5:** Absolute amounts of deposited material after 10 min of electrodeposition in multi-metal electrolytes at different cadmium/nickel ratios. The percentages indicate the amount of the deposited material consisting of dendrites

Rel. amount of Cd to Ni in the electrolyte (%)	No CNF				a-CNF			
	10%	25%	75%	90%	10%	25%	75%	90%
Yield (mg)	52	64	61	55	87	84	65	63
*m*_dendrite_ (mg)	22	25	2	<1	—a	54	15	13
%dendrite	42%	39%	4%	<2%	—a	64%	23%	20%

a The value associated with a-CNF at a Cd-ratio of 10% was omitted when dendrites did not form and small amounts of nickel co-deposited.

The growth of the dendrites varied in extent depending on the presence of the CNF and its relative concentration of cadmium in the solution. For cadmium rich solutions (0.40 M), the relative enhancement in terms of favoring extensive dendrite growth was the largest, resulting in *ca.* 10 times more mass of dendrites formed (see 3rd row, Table 5), *i.e.*, 20 wt% of the deposited mass were dendrites as compared to less than 2 wt% when no cellulose was present. Table 5 additionally shows that as the cadmium concentration decreased in relation to the nickel concentration, the CNF contributed less to the formation of extensively grown dendrites (as compared to when CNF was not present). At 75% (0.33 M) relative content of cadmium to nickel, the amount of cadmium formed into dendrite structures was *ca.* 5 times higher than in absence of CNF (23% compared to 4%, see 3rd row, Table 5). In essence, at all concentration ratios where cadmium was in excess of the nickel, the presence of the cellulose had a marked impact, *i.e.*, in the region where the dendrites hardly formed for the systems without CNF. Table 5 also shows that in the cadmium-nickel regions where dendrites formed regardless of the presence of CNF (≤50/50), significantly more dendrites formed, with the absolute mass of formed dendrites exceeding 100% as the CNFs were present. At a ratio of 25/75 (0.11 M/0.33 M, Cd to Ni), the mass of dendrites formed over 10 min deposition was 54 mg as compared to 25 mg, with and without the CNF present, respectively (see Table 5, 2nd row). Table 5 also show that total amount of deposited material increased with the increasing formation of dendrites, something which was more prominent at the lower Cd/Ni-ratios, where the amount of deposited material could increase with up to 30% (see Table 5, 1st row).

An aspect that is important to highlight is that electrodeposition of cadmium for recycling purposes has to be performed until the concentration reaches a sufficiently low level (<5 μg L^−1^ according to regulatory bodies world-wide^54^) due to the toxic nature of the metal. In leachates containing both nickel and cadmium, co-deposition can become a significant problem when the cadmium concentration has reached such levels. It is therefore highlighted that further research of the effects of CNF in low concentration regimes of cadmium should be made.

## Conclusions

Nanocellulose (CNF) was used as electroactive additive in the deposition and separation of nickel (Ni) and cadmium (Cd) in the recycling of NiCd-batteries. Electrolyte concentrations corresponding to industrially relevant recycling levels (>20 g L^−1^, *ca.* 0.44 M) transformed the Cd-deposits from truncated 25-100 μm metallic dodecahedrons into dendrites growing radially outwards from the collector electrode surface when 0.05 wt% CNF was present. From single-metal solutions, the induced dendritic growth resulted in *ca.* 15% greater yields, as compared to when only the truncated particles formed (when no CNF was used). In comparison, as nickel was deposited at the same 0.44 M concentration, it was concluded that CNF had no effect on the morphology of the deposited material. However, when comparing negatively charged (−75 μeq. g^−1^) with positively charged nanocellulose (+85 μeq. g^−1^), it was concluded that the mass of the deposits was similar for cadmium, whereas the nickel depositions showed 20% smaller mass depositions when using positively charged CNF. This allows for better selectivity in extraction of cadmium from Ni–Cd mixtures, where it is desirable for the Ni to stay in solution. The incorporation of CNF in depositions from mixed metal solutions proved even more effective in increasing the deposition of cadmium by up to 30%. Furthermore, at industrially relevant concentrations, cadmium could be selectively extracted as high purity metallic cadmium (≥99%) with less than 0.005 mol m^−2^ of nickel. Varying Cd/Ni-ratios, ranging from 1 : 9 to 9 : 1, showed that CNF either induced dendritic growth at concentrations where dendritic growth otherwise did not occur, or enhanced the formation of dendritic structures in other cases. The formation of these dendritic structures improves the recycling collection productivity as it creates easily detachable dendritic structures of pure metallic Cd. The detachment of the metallic cadmium structures can be facilitated with simple manual shaking or ultrasound energy.

The extensive variety of cellulosic material modifications with adjustable charge and charge density are herein argued to open up for future studies focusing on the efficacy of anionic as well as cationic cellulose derivatives for selective metal ion electrodeposition.

## Author contributions

B. W. H. carried out all experiments in the article including the isolation and functionalization of the nanocellulose. B. W. H also conducted the characterization of cellulose surface charge and microscopy. O. K carried out the initial electrodeposition experiments, outlining the conditions for the electrodeposition protocols. X. X. performed XRD-measurements. B. W. H. and R. T. O. wrote the manuscript. S. E. M., X. X., Y. L., R. L. A. and A. P. critically reviewed the article and provided assistance with interpreting the findings. R. T. O. recognized the concept of using cellulose nanofibers to affect the deposition of nickel and cadmium during electrodeposition.

## Conflicts of interest

The authors have no conflicts of interests to declare.

## Supplementary Material

NA-005-D3NA00401E-s001
