# Supplementary material for: Cellulose nanofibers (CNFs) in the recycling of nickel and cadmium battery metals using electrodeposition
Source: Nanoscale Adv. 2023 Jul 18;5(19):5263–75. doi: 10.1039/d3na00401e (PMC10521207; doi:10.1039/d3na00401e)
Supplement: NA-005-D3NA00401E-s001 [file NA-005-D3NA00401E-s001.pdf]

Electronic Supplementary Material (ESI) for Nanoscale Advances.
This journal is © The Royal Society of Chemistry 2023

Cellulose Nanofibers (CNFs) in the Recycling of Nickel and Cadmium Battery Metals using Electrodeposition

B. W. Hoogendoorn[a], O. Karlsson[a], X. Xiao[a], A. Pandey[a], S. E. Mattsson[b], V. Ström[c], R. A. Andersson[a], Y. Li[a] and R. T. Olsson[a]

[a.] *Department of Fibre and Polymer Technology, School of Engineering Sciences in Chemistry, Biotechnology and Health, KTH Royal Institute of Technology, SE-100 44 Stockholm, Sweden. E-mail: rols@kth.se;*
[b] *SAFT AB, Jungnergatan 25, 572 32 Oskarshamn, Sweden.*
[c] *Department of Material Science and Engineering, School of Industrial Engineering and Management, KTH Royal Institute of Technology, Brinellvägen 23, SE-100 24. Stockholm, Sweden.*

Table S1: Composition of the electrolytes used during the electrodeposition of nickel and/or cadmium*

Sample**	Electrolyte types	C(Ni^{2+}) [M]	C(Cd^{2+} [M]	C(a-CNF) [g/L]	C(q-CNF) [g/L]
1	Ni-deposition single-metal reference	0.44	0	0	0
2	Ni-deposition single-metal a-CNF	0.44	0	0.5	0
3	Ni-deposition single-metal q-CNF	0.44	0	0	0.5
4	Cd-deposition single-metal reference	0	0.44	0	0
5	Cd-deposition single-metal a-CNF	0	0.44	0.5	0
6	Cd-deposition single-metal q-CNF	0	0.44	0	0.5
7	Ni/Cd-deposition mixed-metal reference	0.22	0.22	0	0
8	Ni/Cd-deposition mixed-metal reference	0.40	0.04	0	0
9	Ni/Cd-deposition mixed-metal reference	0.33	0.11	0	0
10	Ni/Cd-deposition mixed-metal reference	0.11	0.33	0	0
11	Ni/Cd-deposition mixed-metal reference	0..04	0.40	0	0
12	Ni/Cd-deposition mixed-metal a-CNF	0.22	0.22	0.5	0
13	Ni/Cd-deposition mixed-metal a-CNF	0.40	0.04	0.5	0
14	Ni/Cd-deposition mixed-metal a-CNF	0.33	0.11	0.5	0
15	Ni/Cd-deposition mixed-metal a-CNF	0.11	0.33	0.5	0
16	Ni/Cd-deposition mixed-metal a-CNF	0..04	0.40	0.5	0
17	Ni/Cd-deposition mixed-metal q-CNF	0.22	0.22	0	0.5

*All reactions were performed at potentiostatic conditions at 3.5 V, 40 °C, and for 10 min.

** The electrolyte volume and electrolyte concentration for Na_2SO_4, and H_3BO_3 were identical in all electrolytes (0.5 L, 12 g/L and 40 g/L, respectively).

Table S2: Crystallite size of the nickel and cadmium deposits as determined from the three peaks with the highest intensity.*

Sample	Electrolyte types	Peak 1	Peak 2	average
1	Ni-deposition reference	21	15	18±4
2	Cd-deposition reference	65	56	60±6

*For Ni-deposition, the peak 1 and 2 are the ones corresponding to the (111)-, and the (200)-planes, and for the Cd-deposits, these corresponds to the (002)-, and the (101)-planes.

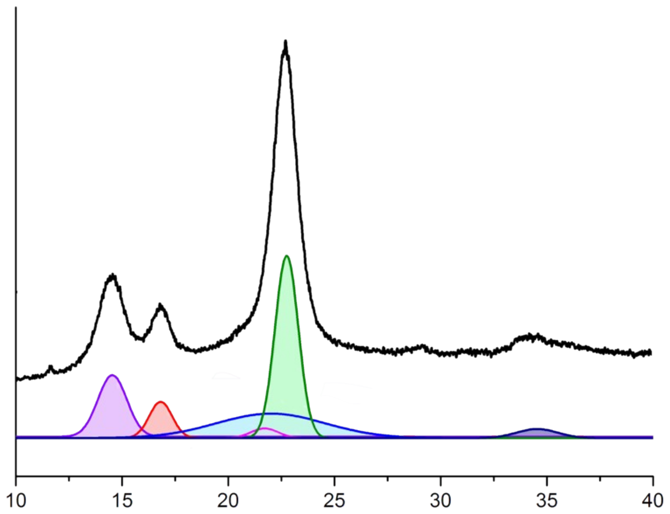

Fig S1. *X-ray diffractogram of the untreated bacterial cellulose and the deconvoluted peaks corresponding to the crystalline peaks corresponding to the (101)- (violet),* $(10\bar{1})$- (red), (021) (purple), and (002)- (green) and (040)-plane (dark blue). The blue curve at *ca.* 21° corresponds to the amorphous contribution obtained through gaussian curve fitting.

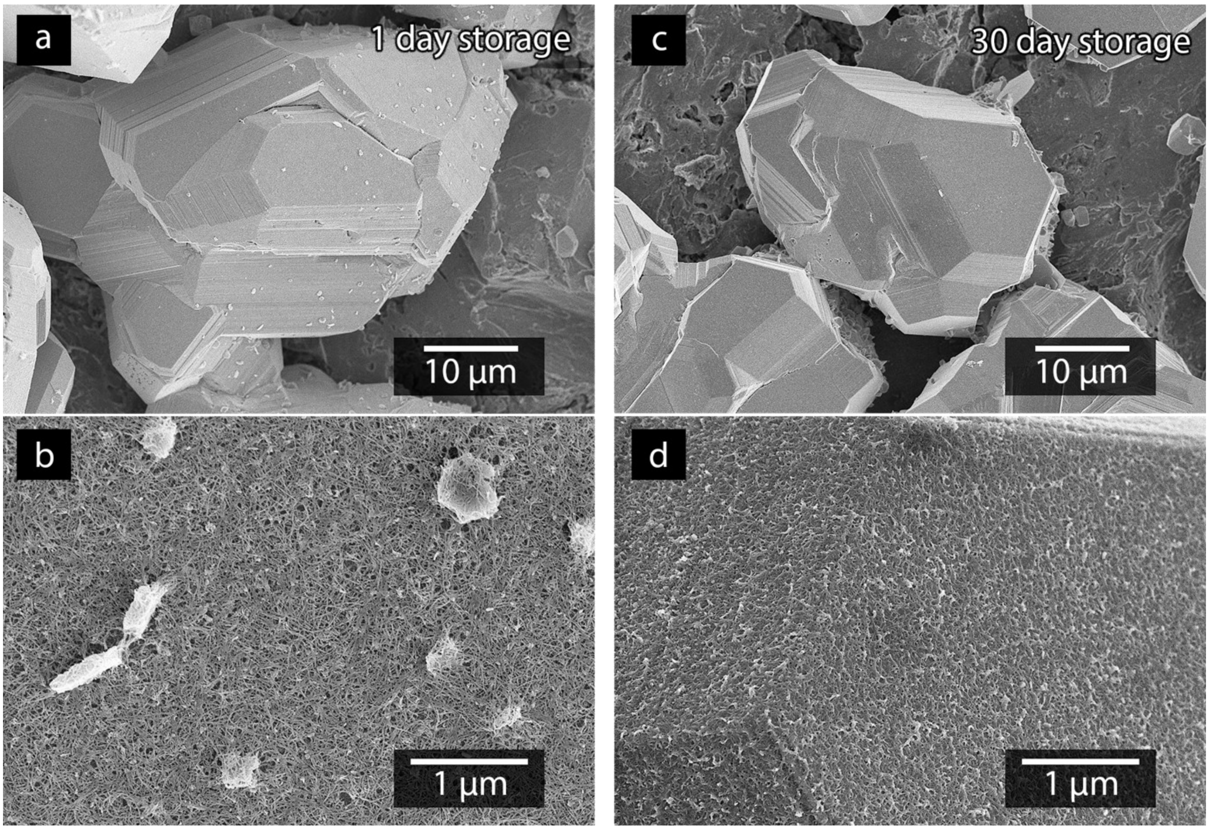

Fig S2. *SEM-micrographs of cadmium deposits one day after electrodeposition (a and b) and 30 days months after electrodeposition (c and d).*

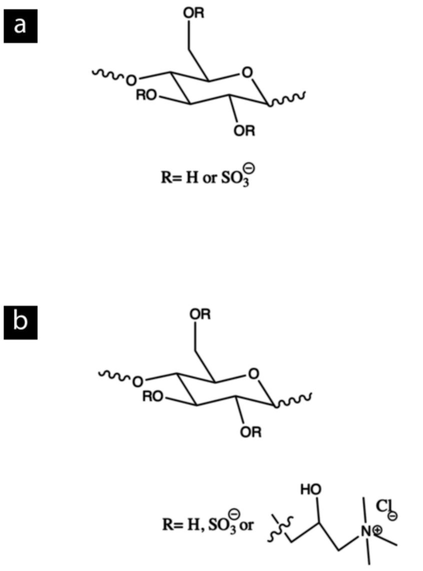

Fig. S3. *Molecular structure of the repeating unit of cellulose after acid hydrolysis (a) and quaternization (b).*

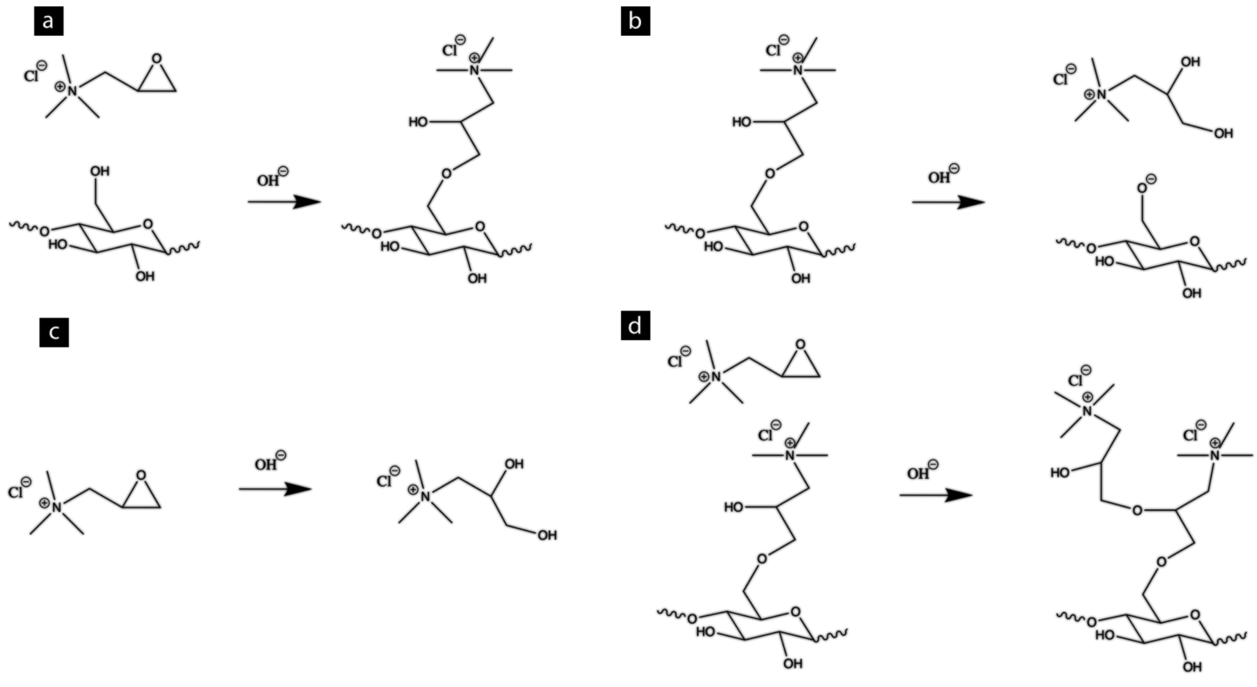

Fig. S4. *Reaction mechanisms for the reactions previously suggested to occur in an aqueous, alkaline medium, including the direct quaternization of cellulose on the hydroxyl group attached to the C_6-atom (a), the degradation of the quaternized cellulose (b), the hydrolysis of the GTMAC-precursor (c), and the repeated substitution occurring on an already quaternized C_6-hydroxyl group. the presence of sulfate half esters has not been taken into account in this schematic.*

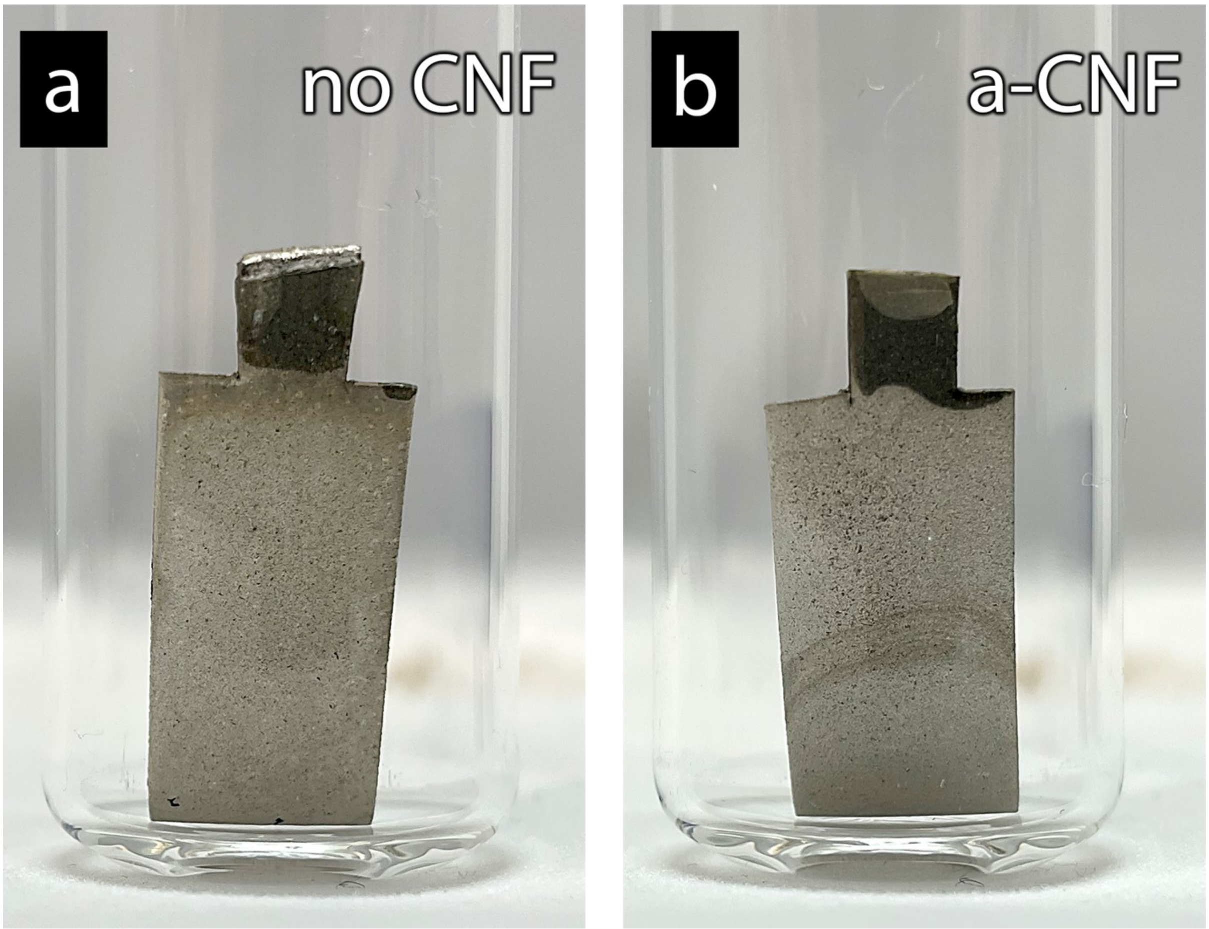

Fig. S5. *Photographs of the active area of electrodes that had been deposited with nickel without the presence of CNF (a) and in the presence of 0.5 g/L of a-CNF (b).*

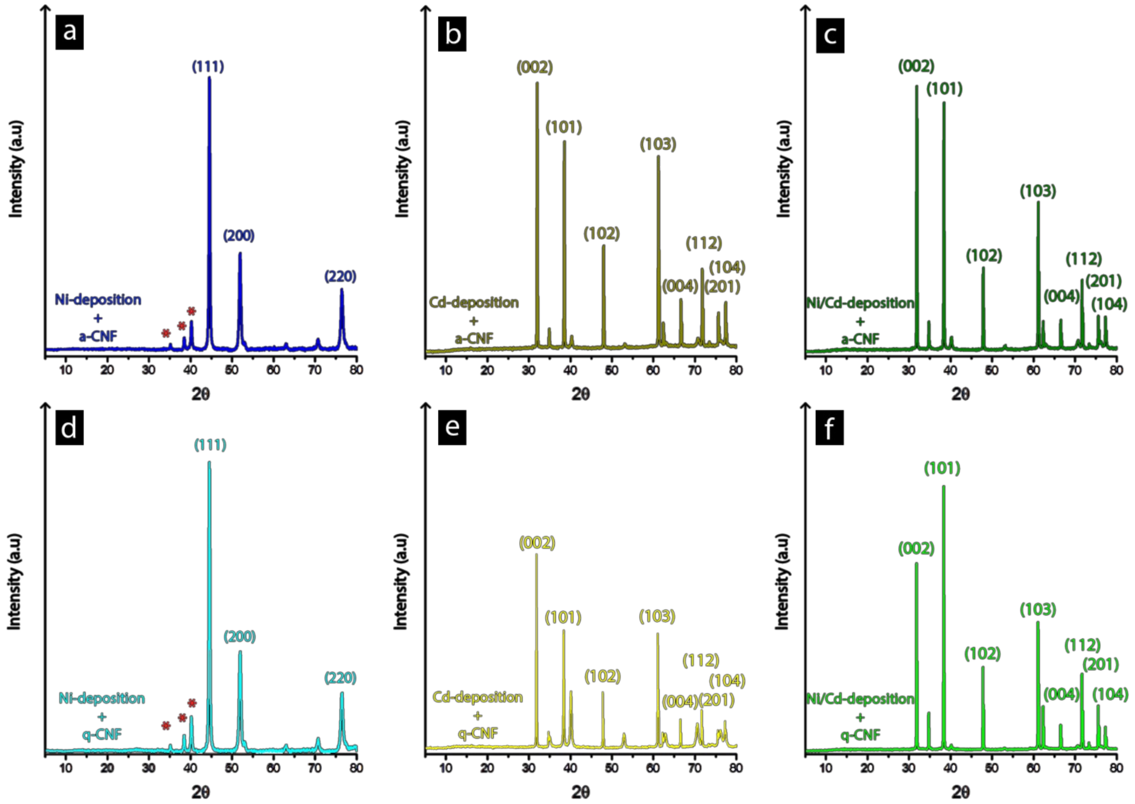

Fig. S6. *X-ray diffractogram of Ti-electrodes after 10 min of electrodeposition in electrolytes containing 0.5 g/L of a-CNF (a-c) or q-CNF (d-f). The electrodeposition protocols were performed in electrolytes containing 0.44 moles/L of NI-ions (a and f), Cd-ions (b and e) or 0.22 moles/L of Ni- and Cd-ions (c and f). The red dots mark the peaks originating from the Ti-electrode.*
